# Chlorine-Induced Toxicity on Murine Cornea: Exploring the Potential Therapeutic Role of Antioxidants

**DOI:** 10.3390/cells13050458

**Published:** 2024-03-05

**Authors:** Seungwon An, Khandaker Anwar, Mohammadjavad Ashraf, Kyu-Yeon Han, Ali R. Djalilian

**Affiliations:** 1 Department of Ophthalmology and Visual Sciences, University of Illinois at Chicago, Chicago, IL 60612, USA; kanwar@uic.edu (K.A.); mashra5@uic.edu (M.A.); biohan72@uic.edu (K.-Y.H.); 2 Clinical Stem Cell Laboratory, UI Blood & Marrow Transplant Program, University of Illinois Hospital and Health Sciences System, Chicago, IL 60612, USA; 3 Department of Pathology, Shiraz University of Medical Sciences, Shiraz 71348-14336, Iran

**Keywords:** chlorine, ROS, JC-1, antioxidants, wound healing

## Abstract

Chlorine (Cl_2_) exposure poses a significant risk to ocular health, with the cornea being particularly susceptible to its corrosive effects. Antioxidants, known for their ability to neutralize reactive oxygen species (ROS) and alleviate oxidative stress, were explored as potential therapeutic agents to counteract chlorine-induced damage. In vitro experiments using human corneal epithelial cells showed decreased cell viability by chlorine-induced ROS production, which was reversed by antioxidant incubation. The mitochondrial membrane potential decreased due to both low and high doses of Cl_2_ exposure; however, it was recovered through antioxidants. The wound scratch assay showed that antioxidants mitigated impaired wound healing after Cl_2_ exposure. In vivo and ex vivo, after Cl_2_ exposure, increased corneal fluorescein staining indicates damaged corneal epithelial and stromal layers of mice cornea. Likewise, Cl_2_ exposure in human ex vivo corneas led to corneal injury characterized by epithelial fluorescein staining and epithelial erosion. However, antioxidants protected Cl_2_-induced damage. These results highlight the effects of Cl_2_ on corneal cells using in vitro, ex vivo, and in vivo models while also underscoring the potential of antioxidants, such as vitamin A, vitamin C, resveratrol, and melatonin, as protective agents against acute chlorine toxicity-induced corneal injury. Further investigation is needed to confirm the antioxidants’ capacity to alleviate oxidative stress and enhance the corneal healing process.

## 1. Introduction

The cornea, as the transparent outermost layer of the eye, is susceptible to injuries and damage from various factors [1,2,3,4,5,6,7]. Corneal wound healing is a crucial process for restoring its integrity and visual function. However, oxidative stress resulting from reactive oxygen species (ROS) can hinder the healing process, leading to delayed recovery and potential complications, such as photokeratoconjunctivities, photokeratitis, pingueculae and pterygia, cataracts, glaucoma, and macular degeneration [2,3,4,5,6,7,8,9,10]. Corneal injuries trigger an inflammatory response, leading to the release of ROS, which can cause cellular damage and impede the healing process [11,12,13]. Oxidative stress disrupts cellular functions, delays the migration and proliferation of corneal cells, and interferes with the extracellular matrix remodeling necessary for effective wound closure [13,14,15]. Antioxidants are molecules that neutralize ROS, preventing cellular damage and maintaining a healthy redox balance [16,17,18,19]. They scavenge free radicals, stabilize cell membranes, and modulate signaling pathways involved in inflammation and tissue repair. In recent years, research has focused on the potential benefits of antioxidants in supporting corneal wound healing [20,21,22,23,24]. The enzymatic antioxidants that have been documented to be present in the cornea include superoxide dismutases (SODs), catalase (CAT), glutathione peroxidases (GPXs), reductase (GR), and glucose-6-phosphate dehydrogenase (G6PD) [25,26].

Common antioxidants include vitamins C and E, superoxide dismutase (SOD), glutathione, and various plant-derived compounds that also have antioxidant effects [27,28]. The therapeutic effects of antioxidants are investigated on corneal epithelial and stromal cells exposed to oxidative stress, resulting in wound healing [20,21,22,23,24]. Antioxidants, such as vitamin A, vitamin C, melatonin, NAC (N-acetylcysteine), and resveratrol, play essential roles in neutralizing harmful reactive oxygen species (ROS) and protecting cells from oxidative damage [25]. Vitamin A, or beta-carotene, is an antioxidant and can be effective in enhancing wound strength in rats [29]. Vitamins C, D, and E and acetylcysteine helped corneal wound healing [30,31,32]. Especially, vitamin C prevents lipid peroxidation and apoptosis in corneal endothelial cells and improves the antioxidant enzyme activity in rat eyes [33,34,35]. Melatonin is a hormone that regulates the sleep–wake cycle and has strong antioxidant properties. Research suggests that melatonin may help protect ocular tissues, including the cornea, from oxidative damage caused by environmental factors such as UV radiation. Melatonin ameliorates oxidative stress in granular corneal dystrophy, dry eye, and diabetic models [36,37,38,39,40,41]. N-Acetylcysteine (NAC) exhibits various beneficial effects, such as rescuing oxidative stress-induced angiogenesis in a mouse corneal alkali-burn model, increasing corneal endothelial cell survival in a mouse model of Fuchs endothelial corneal dystrophy, reducing oxidative stress for cytosine arabinoside in a rat model, and promoting the long-term survival of cones in a model of retinitis pigmentosa [42,43,44,45]. Resveratrol demonstrates protective effects on human corneal epithelial cells, safeguarding them from inflammation, oxidative stress damage, cytotoxicity induced by moxifloxacin and benzalkonium chloride, hyperosmolar conditions, and enhancing wound healing through the attenuation of oxidative stress-induced impairment of cell proliferation and migration, showcasing the potential for the treatment of dry eye disease and various ocular diseases [46,47,48,49,50,51]. Of note, some of the antioxidants noted above, such as resveratrol, may not be strictly antioxidants and may affect other pathways other than ROS activity. 

Several studies have highlighted the significance of antioxidant activities within the cornea. Tsao et al. explored the effect of total antioxidant capacity (TAC) in aqueous humor on corneal endothelial health, discovering that both TAC and ascorbic acid (AA) independently safeguarded against low endothelial cell density [52]. Additionally, Higuchi et al. conducted research into the role of antioxidants in the treatment of corneal disorders, pinpointing selenoprotein P as a substance that imparts antioxidative effects on corneal epithelial cells [53]. In their study, Koskela et al. delved into oxidative stress and protein accumulation in different corneal diseases, identifying that oxidative stress and the activation of the molecular chaperone response were prevalent in keratoconus, macular corneal dystrophy, and Fuchs endothelial corneal dystrophy [54]. Stoddard et al. evaluated the bioavailability and effectiveness of antioxidants in human corneal limbal epithelial cells, ascertaining that quercetin, epigallocatechin gallate, n-propyl gallate, and gallic acid all demonstrated antioxidant activity [55]. These collective studies underscore the vital role of antioxidants in preserving corneal health and their potential in the therapeutic treatment of corneal disorders. 

Acute chlorine toxicity on the cornea refers to the harmful effects of exposure to chlorine gas or chlorine-containing substances on the eye’s corneal tissue [56,57]. Chlorine is a highly reactive and corrosive chemical commonly used in industrial settings, swimming pools, and household cleaning products [57,58,59,60]. When chlorine gas or chlorine-based compounds come into contact with the cornea, they can cause severe damage, leading to various ocular symptoms and potential long-term consequences [57,61,62]. Exposure to chlorine gas can lead to symptom onset at concentrations of 1–3 ppm, which is characterized by mucus membrane irritation. Eye irritation becomes evident at 5–15 ppm, accompanied by moderate upper respiratory tract irritation [56]. Higher concentrations of chlorine gas, such as 430 ppm, can result in death within 30 min, and concentrations exceeding 1000 ppm can lead to death within just a few minutes [56]. The severity of symptoms tends to increase with higher concentrations of chlorine gas [63]. Following chlorine exposure, the eyes may show signs of infections, abrasions, and corrosions in the conjunctiva [64]. The symptoms from chlorine gas exposure can occur immediately or be delayed, appearing 24 h after exposure [56,63]. Chlorine-related corneal injuries typically heal within one to two days and are characterized by a burning sensation and superficial disruption of the corneal epithelium. The cornea is highly sensitive and vulnerable to chemical exposure, and acute chlorine toxicity on the cornea can lead to a range of symptoms reported, including tearing, soreness, severe discomfort, conjunctiva edema, conjunctivitis, excessive tearing, blurred vision, a sensation of having a foreign object in the eye, photophobia, corneal abrasions, and superficial punctate keratopathy [65,66,67,68], as well as foreign body sensation in the eye, pterygium, chronic conjunctivitis, and premature presbyopia [56,61]. The affected eye may also become swollen, and vision may be temporarily blurred or reduced [69,70]. Chlorine exposure can cause direct injury to the corneal epithelial cells, leading to the loss of the epithelial layer and the formation of corneal ulcers [56,71]. Chlorine, a disinfectant used in swimming pools and tap water, can damage the corneal epithelium. As a result, frequent swimmers may experience symptoms such as redness, itching, ocular surface epithelial damage, and eye irritation [71]. An ophthalmic examination may reveal ciliary injection and superficial punctate keratitis, which can be attributed to chlorine’s presence in swimming pools [72].

Our study demonstrates that antioxidants have protective effects on corneal cells, shielding them from oxidative damage caused by Cl_2_ exposure. Furthermore, these antioxidants promote cell migration and accelerate wound closure, indicating their potential to enhance the healing process of corneal injuries. Combining antioxidant treatment with standard care or other regenerative approaches may result in synergistic effects, further augmenting corneal wound healing. Future research should focus on (1) exploring the underlying mechanisms of Cl_2_ injury to the cornea and the protective role of antioxidants in observed changes, such as fluorescein staining, corneal thickness, and epithelial edema; (2) evaluating antioxidants using in vivo and ex vivo models; and (3) optimizing antioxidant formulations, dosages, and delivery methods to maximize their therapeutic potential. Antioxidants show significant promise in supporting corneal wound healing by combating oxidative stress and creating an environment conducive to tissue repair. As our understanding of their mechanisms deepens and more clinical evidence emerges, antioxidant-based therapies could become valuable tools in ophthalmology, contributing to the recovery of corneal injuries and overall improvement of ocular health.

## 2. Materials and Methods

### 2.1. Materials

The following materials were used for this study: vitamin A (#11017, Cayman, CO, USA), vitamin C (#A4403, Sigma-Aldrich, St. Louis, MO, USA), melatonin (#M 5250, Sigma-Aldrich, St. Louis, MO, USA), N-Acetyl Cysteine (NAC; #A9165, Sigma-Aldrich, St. Louis, MO, USA), chlorine (#198016, Thermo Fisher Scientific, Waltham, MA, USA), and resveratrol (#554325, Sigma-Aldrich, St. Louis, MO, USA).

### 2.2. Cell Culture

Human corneal epithelial cell (HCEC) cultures were initiated from cadaver corneas kindly provided by the Illinois (Chicago, IL, USA) and Midwest eye banks (Ann Arbor, MI, USA) [73,74]. Human corneal epithelial cells were cultured in 10% fetal bovine serum (FBS; # F2442, Sigma-Aldrich, St. Louis, MO, USA), 1X L-glutamine (#MT25005CI, Corning, NY, USA), 1X NEAA (#11140050, Gibco, Bilings, MT, USA), and 1% penicillin-streptomycin (P/S; #MT30002CI, Corning, NY, USA) in 5% CO_2_ at 37 °C [73,74].

### 2.3. LDH Toxicity Assay

HCECs were precisely dispensed into 96-well culture plates at a density of 3 × 10^4^ cells per well, utilizing a complete growth medium optimized for this cell type. Following an incubation period of 12 h to permit cell adhesion and stabilization, the cells underwent a single washing step with 200 μL of phosphate-buffered saline (PBS) at isotonic concentration. Subsequently, the cells were subjected to varying concentrations of antioxidant compounds, which were administered in a basal Dulbecco’s Modified Eagle Medium (DMEM) and incubated for 24 h to assess the protective efficacy against oxidative stress. In parallel, to evaluate the cellular response to Cl_2_, a similar protocol was employed wherein post-wash, HCECs were incubated with Cl_2_ at concentrations ranging from 1 to 3000 ppm, dissolved in DMEM, for 24 h. Upon the completion of the exposure period, a volume of 50 μL of the cell culture supernatant was carefully combined with an equal volume of the LDH reaction mixture, prepared by the stipulated guidelines provided by the manufacturer (#C2030, Thermo Fisher Scientific, Waltham, MA, USA). This mixture was then transferred to a 96-well flat-bottom plate and allowed to incubate at ambient temperature for 30 min, facilitating the development of the enzymatic reaction. The resultant chromogenic substrate conversion was quantitatively measured by recording the optical density at dual wavelengths, specifically 490 nm and 680 nm, utilizing a Cytation5 microplate reader. The reliability and reproducibility of the data were ensured by conducting the assays in triplicate across six independent experimental replicates, as substantiated by references [73,74].

### 2.4. Cell Proliferation

HCECs were cultured on a 4-well chamber slide and incubated for 12 h to promote cell adhesion and stabilization. After this, the cells were treated with a different culture medium for 2 h, washed, and then cultured for another 24 h to encourage proliferation. The proliferation of HCECs was assessed by measuring DNA content using the CyQuant^®^ NF Cell Proliferation Assay (#C35006, Invitrogen, Waltham, MA, USA). Following a total of 36 h of incubation, the supernatant was removed, and cells were incubated with a 1× CyQuant dye solution for fluorescence development. Fluorescence intensity, reflecting cell proliferation, was measured with an excitation wavelength of 485 nm and an emission wavelength of 530 nm using a Gen5 plate reader and conducted in triplicate for six samples to ensure data reliability [74].

### 2.5. In Vitro Scratch Assay

HCECs were plated in 6-well culture plates at a density of 5 × 10^6^ cells per well, using media supplemented with 10% FBS to ensure optimal growth conditions. Following a 12-h incubation period to establish confluent monolayers, a sterile 200 μL pipette tip was employed to introduce a standardized scratch, simulating a wound. After this wounding procedure, monolayers were rinsed twice with 1× PBS to eliminate any detached cells. Prior to the administration of antioxidant treatments, cells were subjected to a 30-min exposure to Cl_2_, which was followed by another two 1× PBS washes to remove any residual Cl_2_. The migration and closure of the scratch wound were monitored at designated time intervals. This was accomplished by capturing sequential images of the scratch area with a high-resolution spinning disk confocal microscope (Z1; Carl Zeiss Meditec, Jena, Germany). Quantitative analysis of the wound healing process was facilitated by utilizing ImageJ software to measure the area of the scratch that remained unhealed over time. To ensure the reproducibility and accuracy of the results, these assays were conducted in triplicate with five independent experimental replicates [74].

### 2.6. Mitochondria Membrane Potential Assay

The evaluation of mitochondrial membrane potential in human corneal epithelial cells (HCECs) was conducted utilizing the JC-1 Mitochondrial Membrane Potential Assay Kit (Catalog #ab113850, Abcam, Cambridge, MA, USA), in strict adherence to the supplier’s protocol. HCECs were seeded into 96-well opaque culture plates at a density of 2 × 10^5^ cells per well and allowed to adhere and grow for 24 h. After incubation, the cells underwent a 30-min treatment with Cl_2_, after which they were washed and further incubated with antioxidants for an additional 24 h to assess the protective effects on mitochondrial integrity. The JC-1 assay was then performed by incubating the cells with a 1 µM JC-1 staining solution for 30 min at 37 °C. A parallel set of wells received dilution buffer alone, serving as the control condition. Post incubation, imaging was carried out in the dilution buffer to evaluate the mitochondrial membrane potential. Fluorometric detection was executed employing a Cytation5 plate reader, with an excitation wavelength set at 475 nm and dual emission wavelengths of 530 ± 15 nm and 590 ± 17.5 nm to distinguish between the monomeric and aggregated states of JC-1, respectively, indicative of the mitochondrial membrane potential status [74].

### 2.7. Chlorine Treatment on Naïve Murine Eyes

All in vivo procedures were meticulously executed in strict accordance with the ARVO Statement for the Use of Animals in Ophthalmic and Vision Research, ensuring the highest standards of ethical conduct. The experimental protocol received full endorsement from the University of Illinois at Chicago’s Committee on the Ethics of Animal Experiments (UIC) and the Biosafety Committee, affirming the commitment to ethical research practices. C57BL/6J mice, aged between six and ten weeks, were anesthetized via an intraperitoneal administration of a ketamine–xylazine solution, at dosages of 100 mg/kg and 5 mg/kg, respectively, as referenced in a previous publication [74]. These wild-type mice served as the biological model for assessing ocular toxicity attributable to chlorine exposure. The experimental regimen involved the application of graded concentrations of Cl_2_ (ranging from 1 to 2000 ppm; 10 µL for 30 s) to the murine corneas, administered daily over two weeks. Repetitive measurements were carried out in triplicates with four independent subjects per group. Post-treatment, corneal integrity was evaluated using a 1 mg/mL fluorescein solution (BioGlo; HUB Pharmaceuticals, Plymouth, CA, USA), which was applied to the corneal surface for one minute. After the application, any residual staining solution was carefully blotted away with Kimwipes. The extent of corneal injury was then assessed by examining and capturing images of the fluorescein staining under a Nikon FS-2 slit lamp at 30X magnification. Quantitative analysis of the fluorescein staining intensity was conducted using MetaMorph software (Molecular Devices, Version 7.8.13.0), enabling precise data acquisition on corneal damage following chlorine exposure [74].

### 2.8. Ex vivo Model of Human and Murine Cornea Culture

For murine eyeballs, wild-type mice were used to take basal images of bright-field and fluorescein (1 mg/mL) for basal and followed by treatment with chlorine injury (100–500 ppm, 2 mL, 30–60 min) and then with antioxidants (or vehicle control) for up to 7 days (*n* = 12 per group). The eyes were examined and imaged with a slit lamp every day for 3 days along with fluorescein staining visualized under a cobalt blue light. The outcome measures in the ex vivo human corneas include (i) corneal damage after chlorine exposure (bright-field image and fluorescein staining), (ii) histopathologic examination of the corneal structure and corneal epithelial cells (H&E), and (iii) corneal epithelial/stromal cell apoptosis (TUNEL staining). 

Donated human corneas from an Eversight eye bank facility (Michigan, Ohio, Illinois, New Jersey, and Connecticut) were used. For the human cornea, intact human corneas were selected and washed with 1× PBS containing antibiotics. Human corneas were imaged with bright-field and fluorescein staining (1 mg/mL) for basal and followed by treatment with chlorine injury (100–500 ppm, 2 mL, 30–60 min) and then with treatment with antioxidants (or the vehicle control) for up to 7 days (*n* = 12 per group). The eyes were examined and imaged with a slit lamp every day for 3 days along with fluorescein staining visualized under a cobalt blue light. The outcome measures in the ex vivo human corneas include (i) corneal damage after chlorine exposure (bright-field image and fluorescein staining), (ii) histopathologic examination of the corneal structure and corneal epithelial cells (H&E), and (iii) corneal epithelial/stromal cell apoptosis (TUNEL staining). 

### 2.9. Histology

For hematoxylin and eosin (H&E) staining, cryo-sections were fixed in neutral buffered 10% formaldehyde (Sigma-Aldrich, St. Louis, MO, USA) for 20 min and followed the protocol as previously described [73,74].

### 2.10. Detection of ROS (O_2_^−^ and H_2_O_2_) in Tissues 

For IF staining, two cryo-sections from each group (total of 14 slides, seven different groups) were fixed in neutral buffered 10% formaldehyde (Sigma-Aldrich, St. Louis, MO, USA) for 20 min, following the previously described protocol [73,74]. The final concentrations of DCF-DA (20 μM, Fisher, #50-187-4597) and DHE (5 µM, Cayman, #NC2189794) were freshly prepared and applied to the slides for 30–60 min in a moisture chamber without light. After incubation, the slides were washed twice with 1× PBS and prepared with 1 μL of DAPI for confocal microscopy (Z1; Carl Zeiss Meditec, Jena, Germany). DCF-DA staining was used to detect reactive oxygen species (ROS) in green fluorescence (Ex:Em = 502/523 nm), while DHE staining was specifically used to detect superoxide (O_2_^−^) in red fluorescence (Ex:Em = 490/595 nm).

### 2.11. Statistical Analysis

Statistical analysis was performed utilizing GraphPad Prism 5 software (Version 5.01, GraphPad Software, Inc., San Diego, CA, USA). The data are expressed as mean ± standard deviation (SD), derived from three independent experimental runs. To ascertain the significance of differences between groups, two-tailed nonparametric *t*-tests were employed, with the analyses facilitated by both GraphPad Prism and Microsoft Excel software (Version 2019, Microsoft Corp., Redmond, WA, USA). A P value of less than 0.05 was predetermined as the threshold for statistical significance.

## 3. Results

### 3.1. Cytotoxicity Assay of Antioxidants and Cl_2_ on HCECs

**Figure 1:**
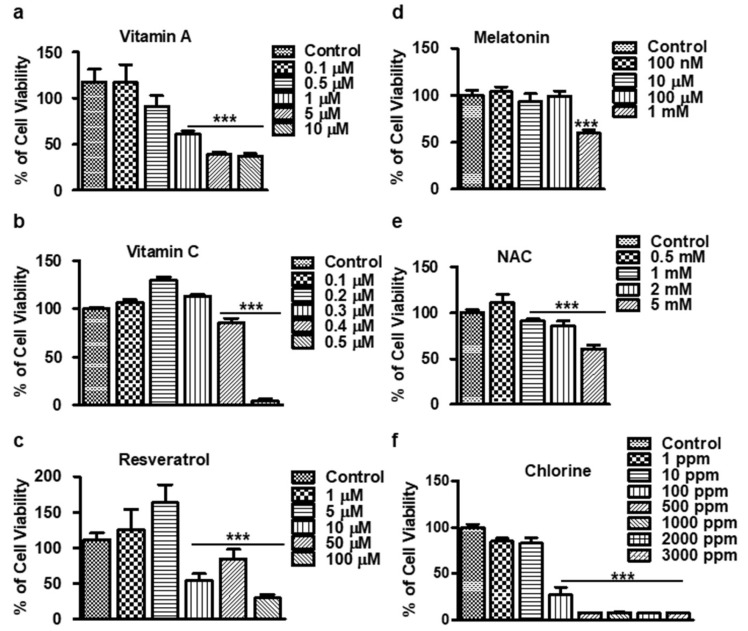
Cytotoxicity assay of antioxidants in the HCECs. (**a**–**f**) The cells were treated for 24 h with varying concentrations. The cell viability is reported as the percentage of the control group (100%). All data are presented as the mean ± SEM (*n* = 6). A significant difference *** *p* < 0.001 using one-way ANOVA analysis witsh Tukey’s post hoc analysis was observed in the percentage of cell viability vs. the control group (untreated). NAC: N-Acetyl Cysteine.

In this study, we aimed to determine the optimal range of antioxidants and the cytotoxicity concentration (CC50) of Cl_2_ in HCECs using a lactate dehydrogenase (LDH) cytotoxicity assay. In order to determine the non-toxic range for the antioxidant, a range of concentration was tested, which showed a dose-dependent decrease in cell viability, with noticeable effects observed at the following concentrations: 0.5 µM for vitamin A, 0.4 µM for vitamin C, 10 µM for resveratrol, 1 mM for melatonin, and 1 mM for NAC (Figure 1a–e). Chlorine exposure demonstrated cytotoxic effects on HCECs, with a reduction in cell viability starting at 1 ppm and approximately 50% loss of viability at 100 ppm (Figure 1f). Based on these findings, we identified the optimal dose of antioxidants to be 100 ppm Cl_2_ for our model of chlorine-mediated cell injury. This concentration strikes a balance between antioxidant protection and chlorine-induced injury, making it suitable for further investigation in our experimental setup.

### 3.2. Cell Proliferation of Antioxidants on Cl_2_-Treated HCECs

**Figure 2:**
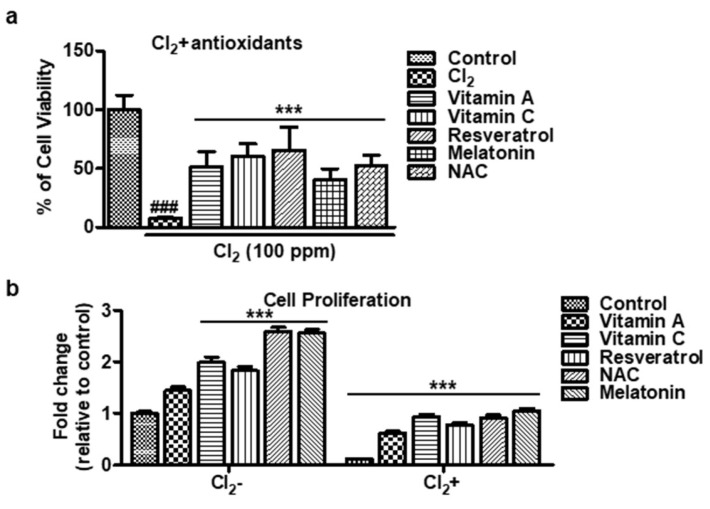
Cell proliferation of antioxidants in Cl_2_-treated HCECs. (**a**,**b**) The HCECs are exposed to 100 ppm Cl_2_ for 30 min and are followed by treatment with antioxidants for 24 h. The results indicate the percentage of cell proliferation vs. the control cells (untreated). Values are the mean ± SEM (*n* = 6). The data were analyzed by one-way ANOVA analysis with Tukey’s post hoc analysis. A significant difference, ### *p* < 0.001 was observed in the percentage of cell viability vs. untreated cells and Cl_2_-treated cells. A significant difference, *** *p* < 0.001 was observed in the percentage of cell viability vs. antioxidant-treated cells. Vitamin A: 100 μM, vitamin C: 300 μM, resveratrol: 5 μM, melatonin: 100 μM, NAC: 500 μM.

We investigated the impact of antioxidants on HCECs after exposure to Cl_2_. At 100 ppm Cl_2_ exposure, cell viability decreased, but treatment with antioxidants (vitamin A, vitamin C, resveratrol, melatonin, and NAC) effectively reversed the Cl_2_-induced cell damage (Figure 2a). Moreover, incubation with antioxidants alone, without Cl_2_ exposure, led to a significant increase in cell proliferation compared to the untreated control (vitamin A: 1.45 ± 0.17, vitamin C: 1.99 ± 0.26, resveratrol: 1.83 ± 0.20, melatonin: 2.59 ± 0.21, NAC: 2.55 ± 0.16 vs. control: 1.0 ± 0.1) in HCECs (Figure 2b). However, when HCECs were exposed to 100 ppm Cl_2_ before antioxidant incubation, the fold change in cell proliferation significantly decreased compared to the untreated control (vitamin A: 0.62 ± 0.08, vitamin C: 0.93 ± 0.13, resveratrol: 0.77 ± 0.10, melatonin: 0.92 ± 0.13, NAC: 1.05 ± 0.11, Cl_2_: 0.12 ± 0.01 vs. control: 1.0 ± 0.1) in HCECs (Figure 2b). These results strongly suggest that antioxidants have the potential to reverse Cl_2_-mediated inhibition of cell proliferation in HCECs. They not only enhance cell proliferation when applied alone but also counteract the negative effects of Cl_2_ exposure, thus offering a promising avenue for mitigating chlorine-induced damage and promoting cell recovery.

### 3.3. Effect of Antioxidant on Cl_2_-Induced ROS Production in HCECs

**Figure 3:**
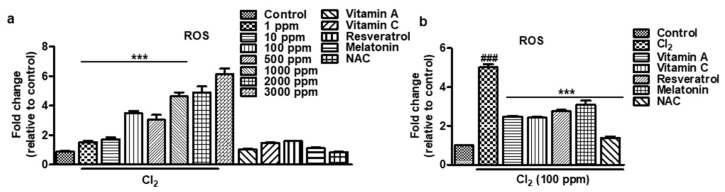
Effect of antioxidants on Cl2-induced ROS production in HCECs. (**a**,**b**) The HCECs were pretreated with Cl_2_ for 30 min followed by 24 h of antioxidant treatment. The results indicate the fold change of ROS level vs. the control cells (untreated). Values are the mean ± SEM (*n* = 6). A significant difference, *** *p* < 0.001 was observed in the fold change of ROS vs. untreated cells and Cl_2_-treated cells. ### *p* < 0.001 was observed in the fold change of ROS vs. untreated cells and antioxidant-treated cells. Vitamin A: 100 μM, vitamin C: 300 μM, resveratrol: 5 μM, melatonin: 100 μM, NAC: 500 μM.

To assess the impact of antioxidants after Cl_2_ exposure on HCECs, we measured cellular ROS levels. As depicted in Figure 3a, various concentrations of Cl_2_ induced a significant four~six-fold increase in cellular ROS accumulation compared to the untreated control (Cl_2_ 1 ppm: 1.53 ± 0.18, 10 ppm: 1.72 ± 0.30, 100 ppm: 3.49 ± 0.36, 500 ppm: 3.04 ± 0.84, 1000 ppm: 4.63 ± 0.67, 2000 ppm: 4.89 ± 1.02, 3000 ppm: 6.14 ± 0.94 vs. control: 0.89 ± 0.04). In contrast, treatment with antioxidants did not show a significant difference compared to the untreated control (vitamin A: 1.01 ± 0.13, vitamin C: 1.47 ± 0.07, resveratrol: 1.61 ± 0.04, melatonin: 1.12 ± 0.10, NAC: 0.85 ± 0.07 vs. control: 0.89 ± 0.04). Moreover, when cells were exposed to 100 ppm Cl_2_, ROS levels surged by four to five times; however, incubating antioxidants mitigated the cellular ROS accumulation to one~three-fold compared to the Cl_2_-treated group. These results highlight that Cl_2_ exposure induces mitochondrial damage and elevates oxidative stress, resulting in a significant increase in cellular ROS levels (Figure 3b). However, the administration of antioxidants effectively combats these deleterious effects, offering a potential therapeutic approach to ameliorate oxidative stress and its detrimental consequences induced by Cl_2_ exposure in HCECs (Figure 3b).

### 3.4. Mitochondrial Membrane Potential in HCECs

**Figure 4:**
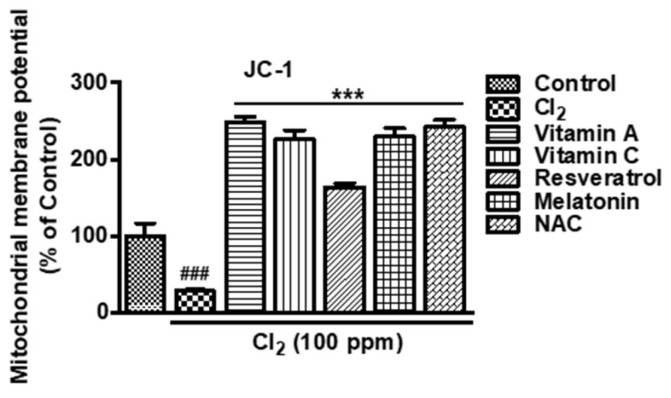
Mitochondrial membrane potential in HCECs. Cells are exposed to 100 ppm Cl_2_ for 30 min prior to treating antioxidants. The results indicate the percentage of mitochondrial membrane potential vs. the control cells (untreated). Values are the mean ± SEM (*n* = 6). A significant difference, ### *p* < 0.001 was observed in the percentage of cell viability vs. untreated cells. A significant difference, *** *p* < 0.001 was observed in the percentage of cell viability vs. Cl_2_-treated cells. Vitamin A: 100 μM, vitamin C: 300 μM, resveratrol: 5 μM, melatonin: 100 μM, NAC: 500 μM.

To assess the effects of antioxidants on maintaining high mitochondrial membrane potential (MMP) after Cl_2_ exposure, HCECs were treated with antioxidants following Cl_2_ exposure. As MMP is a critical indicator of mitochondrial activity, the use of antioxidants appears to preserve mitochondrial function in the presence of Cl_2_. As depicted, exposure to 100 ppm Cl_2_ for 30 min resulted in a significant decrease in the percentage of MMP compared to the control group (100 ppm: 28.59% vs. control: 100%). However, when antioxidants were incubated after Cl_2_ exposure, the decline in MMP induced by Cl_2_ was ameliorated compared to the Cl_2_-exposed group (Figure 4). These findings collectively indicate that Cl_2_ exposure impairs mitochondrial function, leading to a decrease In MMP. However, antioxidant therapy effectively restores the compromised mitochondrial function induced by Cl_2_ exposure, highlighting the potential of antioxidants in preserving mitochondrial activity and mitigating the adverse effects of Cl_2_ on HCECs.

### 3.5. Wound-Healing Assay to Detect Cell Migration of HCECs

**Figure 5:**
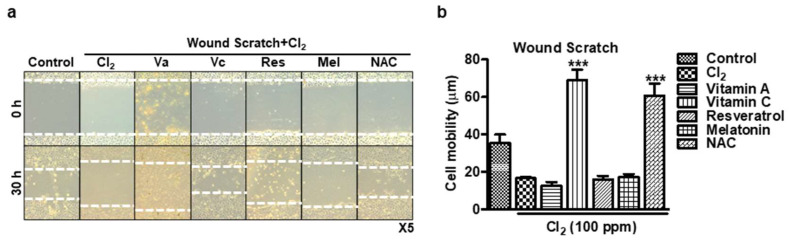
Wound-healing assay to detect cell migration of HCECs. (**a**,**b**) Wound scratch in HCECs exposed to 100 ppm Cl_2_ for 30 min prior to treating antioxidants. (**a**) Representative images showing scratch wound assay in HCLE cells. White dot: wound area. (**b**) Graph showing wound healing rate for different conditions in epithelial scratch wounds (*n* = 5/group) at 30 h. *** *p* < 0.001 was observed in the cell mobility (μm) vs. untreated cells and Cl_2_-treated cells. Va: vitamin A, Vc: vitamin C, Res: resveratrol, Mel: melatonin, vitamin A: 100 μM, vitamin C: 300 μM, resveratrol: 5 μM, melatonin: 100 μM, NAC: 500 μM.

To investigate whether antioxidants can promote wound healing in HCECs delayed by Cl_2_ exposure, we incubated the optimal doses of antioxidants (vitamin A: 100 μM, vitamin C: 300 μM, resveratrol: 5 μM, melatonin: 100 μM, NAC: 500 μM) after Cl_2_ exposure. As depicted in Figure 5, Cl_2_ exposure resulted in cell damage and delayed wound healing, as evidenced by the reduced percentage of wound closure (Cl_2_: 16.6 ± 1.78% vs. control group: 35.4 ± 9.96%). However, when vitamin C and NAC were incubated after Cl_2_ exposure, they significantly promoted wound healing compared to the control group (vitamin C: 68.8 ± 13.09%, NAC: 60.6 ± 14.61%). In contrast, vitamin A, resveratrol, and melatonin did not show a significant improvement in wound healing compared to the control group (vitamin A: 12.6 ± 4.20%, resveratrol: 16 ± 4.14%, melatonin: 17 ± 4.02%). These findings indicate that antioxidant therapy, particularly with vitamin C and NAC, can effectively counteract the delay in cornea cell wound healing caused by Cl_2_ exposure. These antioxidants demonstrate the potential to promote wound closure and may hold promise as therapeutic agents to facilitate the recovery of corneal tissue after Cl_2_-induced damage. Further investigations are warranted to understand the specific mechanisms underlying the wound healing-promoting effects of these antioxidants in HCECs.

### 3.6. In Vivo Evaluation of Cl_2_ Impact on Mice Eyes

**Figure 6:**
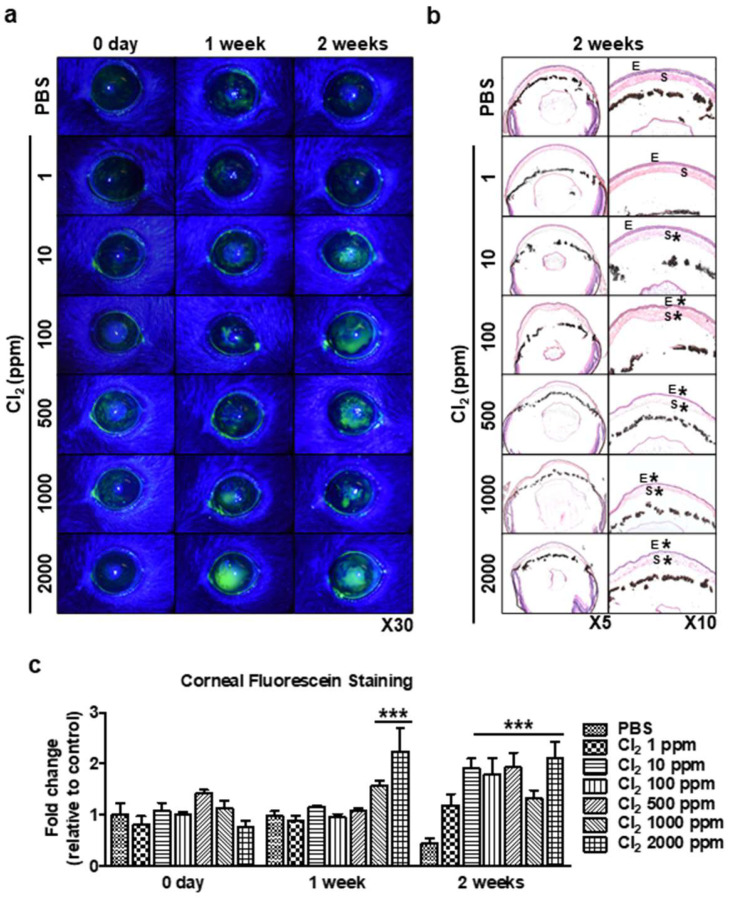
In vivo evaluation of chlorine’s impact on mice eyes using corneal fluorescein staining. Mice corneas were applied to various doses of Cl_2_ (1, 10, 100, 500, 1000, and 2000 ppm; 10 µL, 30 s) once a day for 2 weeks. (**a**) Representative images of murine corneas showing fluorescein staining with Cl_2_ treatment. (**b**) H&E staining of various doses of Cl_2_-treated murine corneas. E: epithelium, S: stroma. *: damaged area. (**c**) Graph showing the intensity fold change of corneal fluorescein staining after application of Cl_2_ treatment (*n* = 4/group) for 2 weeks. Values are the mean ± SEM (*n* = 4). The results indicate that corneal fluorescein staining was greatly increased in a dose-dependent manner compared to the control group (PBS-treated). A significant difference, *** *p* < 0.001 was observed in the fold change of fluorescein staining vs. the control groups (PBS-treated on 1 week or 2 weeks).

Next, we performed experiments in a murine model to determine whether Cl_2_ exposure can cause corneal epitheliopathy in vivo. We applied freshly prepared Cl_2_ and exposure to naïve murine corneas for 1 min per topical application per day for up to 2 weeks. The fluorescein staining was greatly increased at 1 week and 2 weeks compared to day 0 (Figure 6a). Subsequently, Cl_2_ exposure induced damage to the central cornea and stromal layer (asterisk) in murine corneas (Figure 6b). Cl_2_ exposure at 1000 ppm and 2000 ppm resulted in significantly higher corneal fluorescein staining after 1 week compared to baseline (1000 ppm: 1.56-fold, 2000 ppm: 2.23-fold, vs. control). Starting from 2 weeks, Cl_2_ exposure at 10 ppm to 2000 ppm showed a significant increase in corneal fluorescein staining compared to the control group at 2 weeks (10 ppm: 1.9-fold, 100 ppm: 1.79-fold, 500 ppm:1.92-fold, 1000 ppm: 1.4-fold, 2000 ppm: 2.1-fold, vs. control) (Figure 6c). This result indicated that Cl_2_ exposure can cause dose-dependent corneal epitheliopathy in vivo.

### 3.7. Ex Vivo Evaluation of Cl_2_ Effects

**Figure 7:**
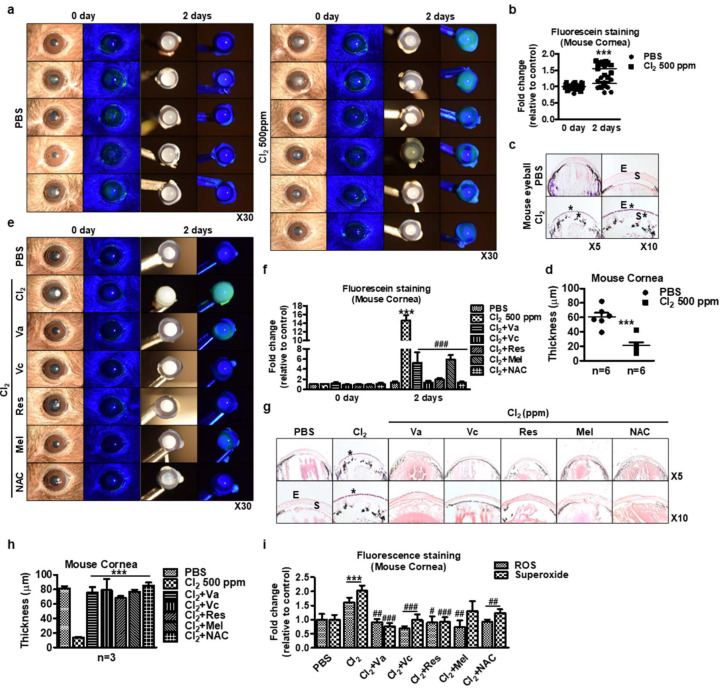
Ex vivo evaluation of chlorine’s effects on mice eyeballs. (**a**–**c**) Mouse eyeballs were exposed to 500 ppm Cl_2_ for 2 h. Subsequently, the eyeballs were washed two times and then incubated in 1x PBS for 2 days. (**a**) Representative images of murine whole eyeballs showing fluorescein staining with or without Cl_2_ treatment. (**b**) Graph showing the intensity fold change of corneal fluorescein staining after application of Cl_2_ treatment (*n* = 12/group) for 2 days. Values are the mean ± SEM (*n* = 11). The results indicate that corneas with 500 ppm Cl_2_ became opaque and hazy with more fluorescein staining of the cornea and conjunctiva than the PBS-treated group. (**c**) H&E staining on murine whole eyeballs after application of PBS or chlorine 500 ppm for 2 days. E: epithelium, S: stroma. *: damaged area. (**d**) Murine cornea thickness after 500 ppm Cl_2_ exposure. *** *p* < 0.001 was observed in the cornea thickness vs. the control group (PBS treatment). Values are the mean ± SEM (PBS: *n* = 11, Cl_2_: *n* = 12). (**e**) Representative images of murine whole eyeballs showed fluorescein staining. (**f**) Graph showing the intensity fold change of corneal fluorescein staining after application of antioxidants (*n* = 4/group) for 2 days. *** *p* < 0.001 was observed in the corneal fluorescein staining vs. the control group (PBS-treated on day 2). ### *p* < 0.001 was observed in the corneal fluorescein staining vs. Cl_2_-treated group on day 2. (**g**) H&E staining on murine whole eyeballs with antioxidants after application of Cl_2_ 500 ppm for 2 days. E: epithelium, S: stroma. *: damaged area. Vitamin A (Va): 100 μM, vitamin C (Vc): 300 μM, resveratrol (Res): 5 μM, melatonin (Mel): 100 μM, and NAC: 500 μM for 2 days. (**h**) Murine cornea thickness. *** *p* < 0.001 was observed in the cornea thickness vs. the control group (PBS-treated). (**i**) Relative fold changes of immunofluorescence intensity from Figure S1c. *** *p* < 0.001 was observed in the ROS and superoxide groups vs. the control group (PBS-treated). **#** *p* < 0.05, **##** *p* < 0.01, **###** *p* < 0.001 were observed in the ROS and superoxide groups vs. the Cl_2_ treatment group.

In parallel experiments, we investigated whether the effect of Cl_2_ exposure on naïve murine cornea was determined by an ex vivo model. Significantly greater corneal fluorescein staining was observed on day 2 following the application of 500 ppm Cl_2_ compared to day 0 (Cl_2_: 1.71-fold vs. PBS: 1.09-fold) (Figure 7a,b). H&E and microscopic analysis of murine eyes showed corneal epithelial loss and stromal edema upon Cl_2_ exposure compared with the control (asterisk, Cl_2_: 20.99 ± 11.16 µm vs. PBS: 60.67 ± 14.23 µm) (Figure 7c,d, Figure S1a,b). Therefore, Cl_2_ exposure to the eye causes severe ocular toxicity, corneal epithelial damage, and abnormal stroma structure ex vivo. As shown in Figure 7e–h, antioxidant-treated groups showed less corneal fluorescein staining (PBS: 1.27-fold, Cl_2_: 14.64-fold, Cl_2_ + vitamin A: 5.23-fold, Cl_2_ + vitamin C: 1.35-fold, Cl_2_ + resveratrol: 1.91-fold, Cl_2_ + melatonin: 5.89-fold, Cl_2_ + NAC: 1.27-fold on day 2) and prevented corneal epithelial loss compared to the Cl_2_-exposed group (PBS: 80.00 ± 2.00 µm, Cl_2_: 14.67 ± 5.51 µm, Cl_2_ + vitamin A: 71.67 ± 15.28 µm, Cl_2_ + vitamin C: 73.33 ± 33.39 µm, Cl_2_ + resveratrol: 66.00 ± 1.73 µm, Cl_2_ + melatonin: 77.33 ± 4.04 µm, Cl_2_ + NAC: 84.00 ± 8.54 µm on day 2). In parallel in vitro experiments, ROS and superoxide were measured to determine the effect of antioxidants after Cl_2_ exposure on the murine cornea. Fluorescence microscopy images showed increasing ROS and superoxide generation after Cl_2_ exposure, while antioxidant treatment ameliorated compared to the Cl_2_-exposed group (Figure 7i, Figure S1c). 

**Figure 8:**
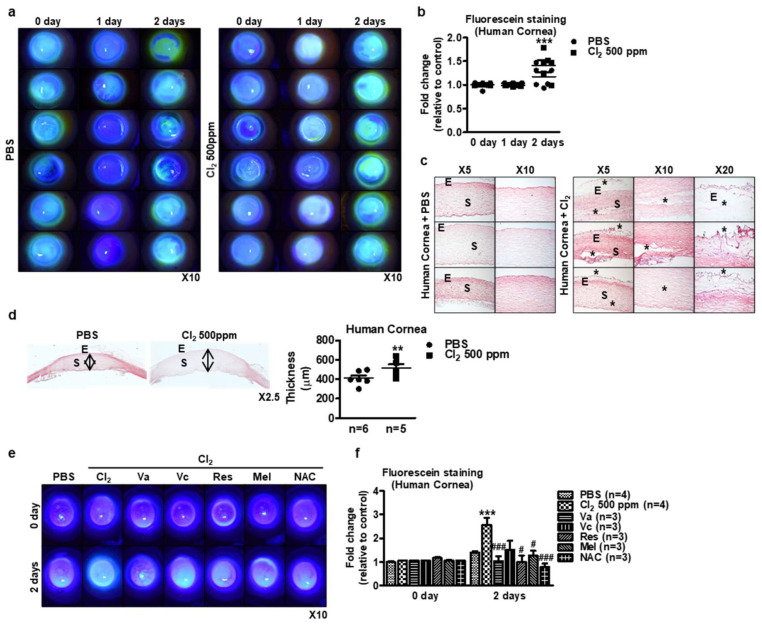
Ex vivo evaluation of Cl_2_ effects on human corneas. (**a**–**d**) Human corneas were exposed to 500 ppm Cl_2_ for 3 days. (**a**) Representative images of human corneas showing fluorescein staining with or without Cl_2_ treatment. (**b**) Graph showing the intensity fold change of human cornea fluorescein staining after Cl_2_ exposure (*n* = 12/group) for 3 days. *** *p* < 0.001 was observed in the fold change of fluorescein staining vs. the control group (PBS). The results indicate that corneas exposure to 500 ppm Cl_2_ showed higher levels of fluorescein staining on both the cornea and conjunctival compared to the PBS-exposed group. (**c**) H&E staining of Cl_2_-treated human corneas. Black star: damaged area, E: epithelium, S: stroma. (**d**) Human cornea thickness after 500 ppm Cl_2_ exposure. ** *p* < 0.05 was observed in the cornea thickness vs. the control group (PBS). Values are the mean ± SEM (PBS: *n* = 12, Cl_2_: *n* = 12). (**e**) Representative images of human corneas showed fluorescein staining by Cl_2_ treatment and followed by antioxidants (Va, Vc, Res, Mel, and NAC). (**f**) Graph showing the intensity fold change of corneal fluorescein staining after application of antioxidants (*n* = 4/group) for 2 days. *** *p* < 0.001 was observed in the corneal fluorescein staining vs. the control group (PBS treatment on day 2). # *p* < 0.05, ### *p* < 0.001 were observed in the corneal fluorescein staining vs. Cl_2_-treated group on day 2.

To investigate the effects of Cl_2_ on human cornea ex vivo, we selected intact human corneas for Cl_2_ exposure. As shown in Figure 8, 500 ppm Cl_2_ exposure was not shown to change corneal fluorescein staining compared to the PBS control group at day 1 (1.03 ± 0.01 vs. 0.98 ± 0.01). Interestingly, Cl_2_ exposure significantly increased corneal fluorescein staining compared to the PBS control group at day 2 (1.45 ± 0.18 vs. 1.07 ± 0.18) (Figure 8b, Figure S2). Subsequently, Cl_2_ exposure induced damage to the central cornea (asterisk), more so than the peripheral cornea or corneal–limbus area in human corneas (Figure 8c). Moreover, H&E and microscopic analysis showed that Cl_2_ exposure resulted in a 1.2~1.6-fold increase in human corneal thickness compared with the control corneas (Figure 8d, Figure S3). In a parallel experiment, antioxidants were treated after Cl_2_ exposure decreased corneal fluorescein staining compared to the PBS control group on day 2 (Figure 8e,f) (PBS: 1.40-fold, Cl_2_: 2.56-fold, Cl_2_ + vitamin A: 1.03-fold, Cl_2_ + vitamin C: 1.50-fold, Cl_2_ + resveratrol: 1.00-fold, Cl_2_ + melatonin: 1.27-fold, Cl_2_ + NAC: 0.78-fold). As a result, Cl_2_ exposure to the cornea showed (1) a rough surface of the epithelial layer on the corneal surface, (2) a loose epithelial layer (corneal epithelial erosion), (3) separation of the epithelial–stromal layer, (4) and corneal edema; however, (5) antioxidant treatment protects Cl_2_-induced epithelial–stromal damage.

## 4. Discussion

In this study, we tested the effects of Cl_2_ in in vitro (human corneal epithelial cells), ex vivo (mouse eyeballs and human corneas), and in vivo mouse models. The main findings of our study are as follows: Cl_2_ exposure significantly (1) decreased cell viability, (2) increased ROS generation, (3) decreased MMP, and (4) delayed in vitro wound healing. However, known antioxidants (vitamin A, vitamin C, resveratrol, melatonin, and NAC) could reverse Cl_2_-mediated damages. Moreover, ex vivo and in vivo studies showed that Cl_2_ exposure showed (5) corneal epithelial damage, (6) separation of the epithelial–stromal layer, and (7) corneal edema. Therefore, we proposed that antioxidants have therapeutic potential to protect against Cl_2_ eye injury and could be used in the development of targeted ocular therapies.

Previous studies have established the toxic effects of chlorine gas exposure. Cl_2_ gas, a highly reactive and toxic substance, is classified as a pulmonary irritant. It finds widespread use in various industries and household applications, including water treatment, disinfection, and cleaning products [59,60,66]. Exposure to chlorine gas, being water-soluble, can result in a range of health issues, contingent upon the dose and duration of exposure [57,59,60]. Acute exposure to high doses of Cl_2_ gas may cause dyspnea, violent cough, nausea, vomiting, lightheadedness, headache, chest pain, abdominal discomfort, and corneal burns. Moreover, even low doses of Cl_2_ gas can lead to chest pain, cough, sore throat, and hemoptysis [59,60,66].

We have previously reported that nitrogen mustard similarly induced ROS, change in MMP, and delay in wound healing in corneal epithelial cells, which was mitigated by the mesenchymal stem cell secretome [74,75]. Our current findings revealed that Cl_2_ decreased cell viability (Figure 1f) and cell proliferation (Figure 2), increased intracellular ROS generation (Figure 3), decreased MMP in HCECs (Figure 4), and delayed wound healing (Figure 5). However, the optimized dose of antioxidants (vitamin A, vitamin C, resveratrol, and melatonin) incubation showed significantly reversed Cl_2_-mediated cellular damages, such as increased cell viability and cell proliferation, downregulated ROS accumulation, and stabilized MMP levels. While we did not investigate the mechanisms underlying the protective effects of antioxidants in our current study, considering our current focus on the therapeutic potential of antioxidants on Cl_2_ exposure, it will be of great interest to investigate the Cl_2_-involved mechanism by dose and exposure duration in the future. 

Our in vitro similarly showed that Cl_2_ exposure delayed corneal wound healing (Figure 5) and increased corneal fluorescein staining in an in vivo model (Figure 6). Interestingly, more than 10 ppm Cl_2_ gradually increased corneal fluorescein staining over a period of two weeks (Figure 6a,c), which indicates a more long-lasting effect. These results suggest that continuous Cl_2_ exposure causes ocular surface epithelial damage and likely deeper layers of the epithelium, including the more basal cells, which include stem/progenitor cells. Only vitamin C and NA incubation after Cl_2_ exposure promotes wound healing in vitro (Figure 5), and other tested antioxidants did not have the same effect on wound healing. It is interesting to note that both vitamin C and NAC are mostly known as antioxidants, while the tested chemicals (vitamin A, melatonin, resveratrol) are also known to affect other pathways. Future studies are needed to determine the effects of antioxidants and the specific mechanisms after Cl_2_ injury.

In murine corneas, Cl_2_ exposure significantly increased epithelial edema (Figure 7c,d). The effect of antioxidants after Cl_2_ exposure in murine corneas in vivo will be studied in the future. Also, our previous study demonstrated that induced loss of membrane integrity of surface epithelium and corneal stromal matrix by nitrogen mustard exposure resulted in epithelial and stromal inflammation and apoptosis [74]. In our study, we employed both in vivo and ex vivo models using human and mouse corneas. To evaluate in vitro data, we conducted ex vivo experiments using both human cornea and mouse cornea. Cl_2_ injury increased mouse corneal fluorescein staining (epithelial damage); however, the antioxidant treatment showed significantly less staining than the Cl_2_-treated group (Figure 7e,f). In correlation with staining data, H&E staining data suggested that Cl_2_ injury significantly damaged the corneal epithelial layer, but antioxidant (Va, Vc, Res, Mel, and NAC) treatment prevented Cl_2_-induced corneal epithelial damage (Figure 7g,h). According to the data, there is a correlation between ROS accumulation in human corneal epithelial cells (Figure 3b) and the effect of antioxidants on MMP (Figure 4). IF staining data further supports the impact of antioxidants on Cl_2_-induced ROS and superoxide detection in in vivo tissues (Figure 7i and Figure S1c). The provided data confirms that staining with ROS and superoxide can ascertain the injury of murine corneal epithelium and the therapeutic effect of antioxidants. 

In parallel experiments, murine eyeballs and donated intact human corneas were used to determine the effects of Cl_2_ in ex vivo conditions. The results showed that corneas exposed to Cl_2_ exhibited increased fluorescein staining on both the cornea and conjunctiva areas (Figure 8a,b). Additionally, H&E staining revealed damaged areas in the Cl_2_-treated human corneas, specifically in the epithelium and stroma. Furthermore, the corneas exposed to 500 ppm Cl_2_ showed increased epithelial edema compared to the control group (Figure 8c,d). Statistical analysis indicated significant differences in fluorescein staining intensity and cornea thickness between the Cl_2_-exposed group and the control group (Figure 8d). More interestingly, antioxidant-treated groups (Va, Vc, Res, Mel, and NAC) decreased fluorescein staining intensity means preventing human corneal epithelial damage compared to Cl_2_-treated human corneas (Figure 8e). It suggests that antioxidants can protect against chlorine exposure to mouse corneas. These samples can be indirectly assessed for antioxidant effects through oxidative stress and superoxide staining [74]. Direct assessment can be achieved using the Trolox Equivalent Antioxidant Capacity method [52,76,77]. Furthermore, we hypothesize that Cl_2_-induced ROS triggers mitochondrial dysfunction. To investigate this, we propose using cellular oxygen consumption rate (OCR) and extracellular acidification rate (ECAR) as functional assays. These tools will allow us to evaluate the capacity of antioxidants to mitigate potential mitochondrial dysfunction caused by Cl_2_-induced ROS. Based on the findings, further investigations and future plans could include as follows: (1) extending the exposure time beyond 3 days to evaluate the long-term impact of Cl_2_ on human corneal tissues; (2) conducting experiments with varying concentrations of Cl_2_ to assess the dose-dependent effects on human corneas; (3) exploring the underlying mechanisms of Cl_2_ injury on the corner and the protection mechanism of antioxidants responsible for the observed changes in fluorescein staining, corneal thickness, and epithelial edema; (4) evaluation of antioxidants in the in vivo and ex vivo models (relating to oxidative stress/superoxide), MMP, Trolox Equivalent Antioxidant Capacity, cellular oxygen consumption rates and extracellular acidification rates, (5) influence of Cl_2_ on the corneal limbal stem cells using 2 mm or limbus-to-limbus corneal wound model with an especially low dose of Cl_2_ treatment; and (6) assessing the effectiveness of potential therapeutic agents or treatments aimed at mitigating Cl_2_-induced corneal damage. It is essential to continue research in this area to enhance our understanding of Cl_2_-induced corneal toxicity and develop strategies to protect and treat affected individuals effectively.

In summary, our study demonstrates the protective role of antioxidants in preventing Cl_2_-induced corneal injury. This protection is associated with enhanced corneal epithelial cell migration, proliferation, and maintenance of mitochondrial dynamic balance in human cornea cells. Furthermore, we successfully replicated Cl_2_-induced corneal injury in both murine eyeballs and donated human corneas using ex vivo models. However, to gain a comprehensive understanding of the effects of antioxidants on Cl_2_-induced injury in the ex vivo model, further studies are warranted.

## 5. Conclusions

Our study sheds light on the potential benefits of antioxidant therapy in countering acute chlorine-induced corneal injury. These findings hold promise for developing effective treatments to safeguard ocular health and mitigate the harmful effects of Cl_2_ exposure to the cornea.

## Data Availability

Data are contained within the article and Supplementary Materials.

## Supplementary Materials

The following supporting information can be downloaded at: https://www.mdpi.com/article/10.3390/cells13050458/s1, Figure S1: H&E staining on Cl_2_-exposed murine eyeball. (a,b) H&E staining of Cl_2_-exposed murine eyeballs for 3 days. (c) IF staining of ROS (DCF-DA: Green), superoxide (DHE: Red), and DAPI (Blue). Scale bar, 50 µm; Figure S2: Fluorescein staining on the human cornea after Cl_2_ exposure. (a,b) Representative images of human corneas showing fluorescein staining with or without 500ppm Cl_2_ treatment for 3 days. BF: Bright field, Flu: Fluorescein; Figure S3: H&E staining on Cl_2_-exposed human corneas. (a,b) H&E staining of Cl_2_-treated human corneas. E: Epithelium, S: Stroma, En: Endothelium.
