# Supplementary material for: Chlorine-Induced Toxicity on Murine Cornea: Exploring the Potential Therapeutic Role of Antioxidants
Source: Cells. 2024 Mar 5;13(5):458. doi: 10.3390/cells13050458 (PMC10930774; doi:10.3390/cells13050458)
Supplement: Supplementary file 1 [file cells-13-00458-s001.zip › cells-2819559-supplementary.pdf]

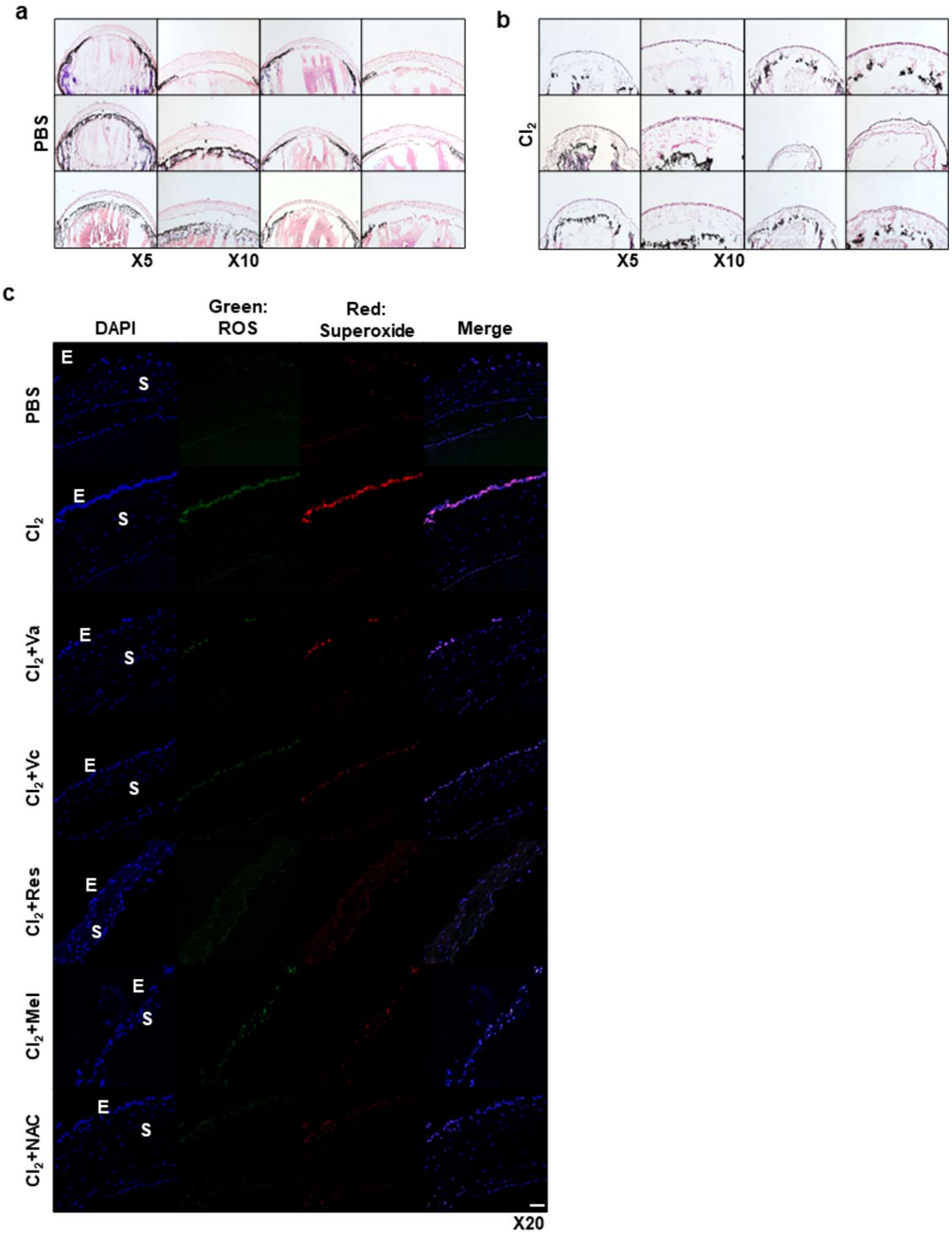

Figure S1. H&E staining on Cl_2-exposed murine eyeball. (a-b) H&E staining of Cl_2-exposed murine eyeballs for 3 days. (c) IF staining of ROS (DCF-DA: Green), superoxide (DHE: Red), and DAPI (Blue). Scale bar, 50 μm.

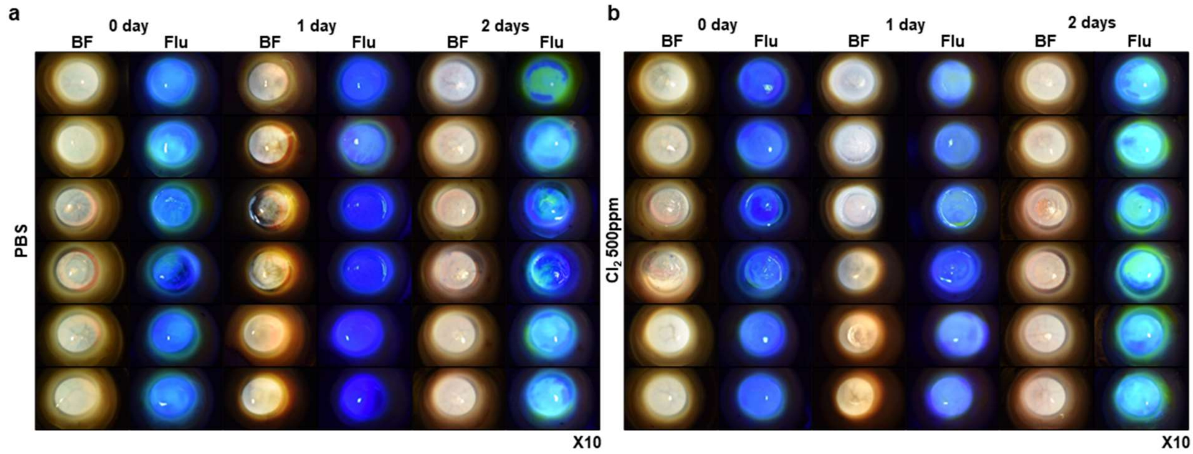

Figure S2. Fluorescein staining on the human cornea after Cl_2 exposure. (a-b) Representative images of human corneas showing fluorescein staining with or without 500ppm Cl_2 treatment for 3 days. BF: Bright field, Flu: Fluorescein.

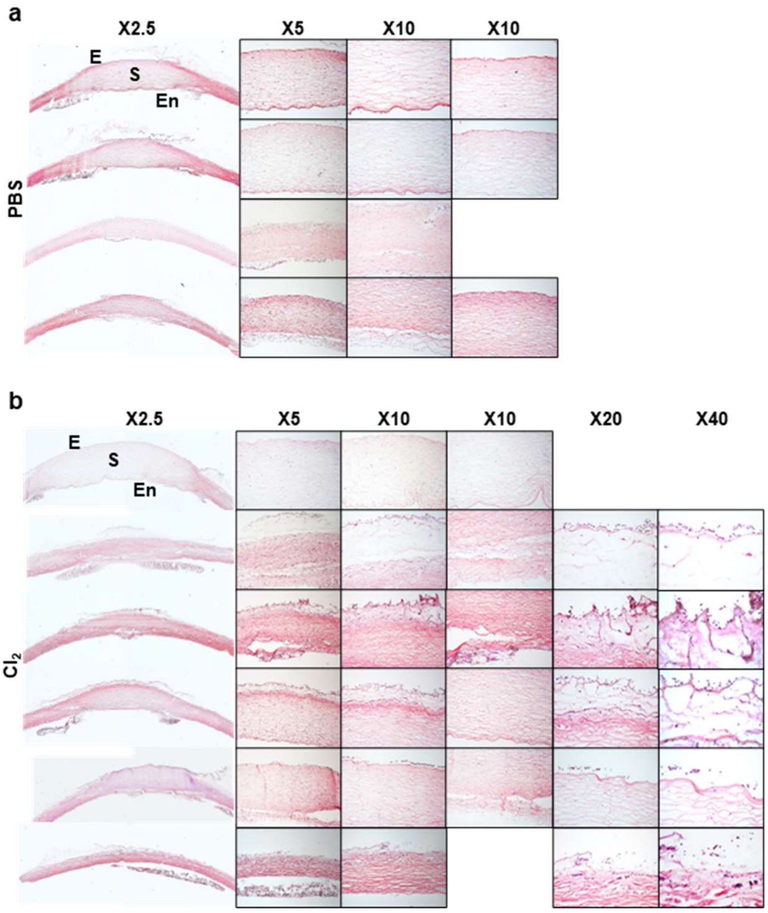

Figure S3. H&E staining on Cl_2-exposed human corneas. (a-b) H&E staining of Cl_2-treated human corneas. E: Epithelium, S: Stroma, En: Endothelium
